# Cyclooxygenase-2 selective inhibition with NS-398 suppresses proliferation and invasiveness and delays liver metastasis in colorectal cancer

**DOI:** 10.1038/sj.bjc.6601489

**Published:** 2004-02-03

**Authors:** M Yao, E C Lam, C R Kelly, W Zhou, M M Wolfe

**Affiliations:** 1 Section of Gastroenterology, Boston University School of Medicine and Boston Medical Center, 650 Albany Street, Boston, MA 02118, USA

**Keywords:** colorectal cancer, COX-2 inhibition, invasiveness, metastasis

## Abstract

Nonsteroidal anti-inflammatory drugs (NSAIDs) have been reported to reduce the risk and mortality of colorectal cancer (CRC) by inhibiting the activity of cyclooxygenase (COX). The present studies were directed to determine whether selective COX-2 inhibition reduces CRC tumour cell proliferation and invasion/migration, and the possible cellular and molecular mechanisms involved. The MC-26 cells are a highly invasive mouse CRC cell line expressing COX-2 protein. NS-398 (100 *μ*M), a highly selective COX-2 inhibitor, decreased cell proliferation by ∼35% of control, as determined using [^3^H]-thymidine incorporation. This reduction in cell proliferation was associated with decreased expression of cyclin D1 and proliferating cell nuclear antigen (PCNA). Furthermore, NS-398 inhibited cell invasion/migration through Matrigel extracellular matrix components at 24 h by ∼60%. The addition of exogenous prostaglandin E_2_ partially attenuated the inhibition of cell invasion by 10 *μ*M NS-398, but failed to reverse the effect of 100 *μ*M NS-398. Matrix metalloproteinases-2 (MMP-2) and -9 (MMP-9) are two enzymes that facilitate cell invasion/migration by degrading the extracellular matrix. In the presence of 100 *μ*M NS-398, Western blot hybridisation analysis and zymography demonstrated that both MMP-2 and MMP-9 protein levels and enzyme activity were decreased by ∼25–30%. In separate studies, NS-398 also inhibited tumour growth *in vivo* and retarded the formation of liver metastasis. The results of these studies indicate that the expression and activity of COX-2 appear to be associated with both the proliferative and invasive properties of CRC. Cyclooxygenase-2 inhibition suppresses tumour cell growth and invasion/migration, and retards liver metastasis in a mouse colon cancer model, via multiple cellular and molecular mechanisms.

Despite significant improvements in its prognosis due to advances in diagnosis and therapy modalities, colorectal cancer (CRC) remains second only to lung cancer as a cause of death from malignant disease in the United States (Landis *et al*, 1998; Brenner, 2002; Singh *et al*, 2002). Epidemiological studies have demonstrated a 40–50% reduction in mortality from CRC in individuals taking nonsteroidal anti-inflammatory drugs (NSAIDs) (Martinez *et al*, 1995; Ritland *et al*, 1999; Smalley *et al*, 1999; Garcia-Rodriguez and Huerta-Alvarez, 2001). These agents appear to reduce the risk of CRC by inhibiting cyclooxygenase (COX), also known as prostaglandin G/H synthase (PGHS), a key enzyme involved in the conversion of arachidonic acid to tissue-specific prostaglandins. Two COX isoforms of cyclooxygenase (COX-1 and COX-2) have been identified, and both isoenzymes are regulated in a different manner and exhibit distinctive functional differences. Cyclooxygenase-1 is expressed constitutively in many cell types, whereas COX-2 is a primary response gene whose expression may be induced by trauma, growth factors, tumour promoters and cytokines (Taketo, 1998a, 1998b; Dannenberg and Zakim, 1999; Williams *et al*, 1999). Cyclooxygenase-2 was first discovered as an oncogene-responsive isoenzyme, and increased COX-2 expression has been found in up to 85% of colorectal adenocarcinomas, while it is undetectable in normal intestinal mucosa (Giardiello *et al*, 1995; Kargman *et al*, 1995).

Nonselective COX inhibitors, which inhibit both isoforms, have been reported to prevent tumorigenesis (Peura, 2002; Schwartz *et al*, 2002). However, the use of nonselective COX inhibitors is often associated with gastrointerestinal (GI) adverse events, whereas COX-2 selective inhibitors are thought to exert anti-inflammatory and their antineoplastic properties with diminished toxicity (Hirata *et al*, 1997; Wolfe, 1998; Wolfe *et al*, 1999; Lichtenstein and Wolfe, 2000). NS-398, *N*-[2-(cyclohexyloxy)-4-nitrophenyl]-methanesulphonamide, is a sulphonamide derivative that inhibits COX-2 specifically, with an IC_50_ of 30 nM. This agent does not affect COX-1 activity at concentrations exceeding 100 *μ*M, and it inhibits COX-1 prostanoid production only minimally, even at a dose exceeding 200 mg kg^−1^ (Futaki *et al*, 1994; Gierse *et al*, 1995). NS-398 can inhibit either chemically induced tumorigenesis in the colon or human cancer xenograft in nude mice (Yoshimi *et al*, 1997, 1999; Sawaoka *et al*, 1998).

NASIDs appear to exert their beneficial effects via multiple mechanisms in cancer chemoprevention. It has been generally assumed that the antiproliferative effects of NASIDs are dependent upon inhibition of COX activity and prostaglandin synthesis. The effects of COX-2 inhibitors on colonic carcinogenesis, cell cycle control, differentiation, angiogenesis and apoptosis in cancer chemopreventation have been described in numerous studies (Tsujii and DuBois, 1995; Tsujii *et al*, 1998). Relatively little attention has been given to COX with relation to tumour cell invasive potential and metastasis. We have recently reported that the COX-2 selective inhibitor rofecoxib decreased cell growth by several mechanisms and decreased the metastatic potential of CRC in the mouse (Yao *et al*, 2003). The mechanisms by which selective COX-2 inhibition inhibits metastasis have not been determined. The present studies were designed to extend our previous studies to determine whether selective COX-2 inhibition with NS-398 likewise reduces CRC tumour cell proliferation and invasion/migration, and to elucidate the possible cellular and molecular mechanisms involved. The results of these studies indicate that the expression and activity of COX-2 appears to be associated with both the proliferative and invasive properties of CRC. Cyclooxygenase-2 inhibition significantly inhibits CRC growth and invasion/migration, and retards the formation of liver metastasis by multiple cellular and molecular mechanisms.

## MATERIALS AND METHODS

### Cell culture

The transplantable mouse CRC cell line MC-26 (Singh *et al*, 1986; Walker *et al*, 1986) was obtained from Dr KK Tanabe (Massachusetts General Hospital, Boston, MA, USA). MC-26 cells were maintained in Dulbecco's modified Eagle's medium (DMEM; Life Technologies, Inc, Gaithersburg, MD, USA) supplemented with 10% foetal calf serum plus antibiotics at 37°C in a humidified atmosphere of 95% air/5% CO_2_.

### Cell proliferation

Cell proliferation was determined by DNA synthesis, and then confirmed by cell number counting. Cells (10^4^ ml^−1^) were seeded onto 12-well plates for 24 h, followed by the addition of different concentrations of NS-398 (Cayman chemical, Ann Arbor, MI, USA). NS-398 was dissolved in dimethylsulphoxide (DMSO) as a stock solution. DNA synthesis was estimated by [^3^H]-thymidine incorporation into cellular DNA. [^3^H]-thymidine 1 *μ*Ci ml^−1^ (New England Nuclear Products, Boston, MA, USA) was added and allowed to label for 6 h at 37°C. Cells were washed × 3 with cold phosphate-buffered saline (PBS), and cold 10% trichloroacetic acid (TCA) was added to cells for 30 min at 4°C. Cells were washed again × 3 with cold PBS, after which they were lysed in 0.1 N NaOH/10% SDS and radioactivity measured in a liquid scintillation counter. Data were expressed as the percentage of control, in order to reduce variation among separate experiments. Protein synthesis was measured by a BCA protein assay kit (Pierce chemical, Rockford, IL, USA), according to the protocol provided by the manufacturer. For cell number counting, cells (10^4^ ml^−1^) were seeded onto six-well plates for 24 h, followed by the addition of different concentrations of NS-398. Cells were harvested and then cell numbers were manually counted under the microscope.

### Prostaglandin E_2_ assay

Prostaglandin E_2_ (PGE_2_), the major metabolite of arachidonic acid metabolism, was measured by ELISA (Cayman Chemical, Ann Arbor, MI, USA) with conditional cell culture medium, according to the protocol provided by the manufacturer. Measurements were made in triplicate in separate experiments.

### Cell invasion/migration assay

A membrane invasion culture system (Collaborative Biomedical Products, Bedford, MA, USA) was used to quantify cell invasion and migration, as described previously (Rozic *et al*, 2001). The upper surface of the polycarbonate filter with 8-*μ*m pores was coated with Matrigel, and placed between the upper and lower well plates of the membrane invasion system. Serum-free Swiss 3T3 fibroblast conditional medium (CM) was prepared by incubation of these cells for 24 h. This medium was used as a chemoattractant in the lower chamber. After reaching 60–70% subconfluence, cells were harvested by trypsinisation and suspended in DMEM, and harvested cells (10 000 ml^−1^) were seeded into the upper chambers. Cells were cultured in the presence of various concentrations of NS-398 alone, or in combination with exogenous PGE_2_ (Cayman Chemical, Ann Arbor, MI, USA) for 24 h. At the end of 24 incubation at 37°C, the cells in the upper surface of the Matrigel-coated filter were mechanically removed, the filters fixed with methanol, stained with haematoxylin and eosin, and the cells on the lower surface were counted manually.

### Gelatin zymography

Gelatin zymography was performed using CM harvested from MC-26 cells under various conditions as described (Nomura *et al*, 1995; Liabakk *et al*, 1996). Conditional medium was collected and concentrated six-fold using Centricon 10 filter (Amicon), and then subjected to 10% SDS–PAGE with 1 mg ml^−1^ gelatin incorporated into the gel mixture. Following electrophoresis at 4°C, the gel was soaked in 2.5% Triton X-100 to remove the SDS, rinsed in H_2_O × 3, and then transferred to a developing buffer (50 mM Tris-HCL pH 7.5, 5 mM CaCl_2_, 200 mM NaCl) at 37°C overnight. Gels were stained with Coomassie blue for 2–3 h and then destained with 30% methanol and 10% acetic acid. The unstained bands (digested gelatin) correspond to the presence of matrix metalloproteinase (MMP) proteolytic activity.

### Animal experimental design

Male BALB/*c* mice, 6-week old, were obtained from Taconic (Germantown, NY, USA). The mouse colon cancer model was established as described previously (Yao *et al*, 2002). MC-26 cells were harvested from subconfluent cultures by exposure to trypsin-EDTA (Life Technologies, Inc, Gaithersburg, MD, USA) for 3 min, centrifugation at 300 **g** for 15 min at room temperature, and then resuspension in serum-free DMEM or Hank's balanced salt solution (Life Technologies, Inc, Gaithersburg, MD, USA), to yield a final concentration of 10^5^ cells ml^−1^. Using a 27-gauge needle and a 1-ml syringe, 100 *μ*l of tumour cell suspension was injected subcutaneously into the flank of the mice. All animal studies were conducted using a protocol approved by the Institutional Animal Care and Use Committee of Boston University Medical Center.

NS-398, dissolved in DMSO, was administered by oral gavage once daily. Mice were randomly divided into three groups (10 animals/group) on day 0 after tumour cell implantation: vehicle (DMSO), NS-398 1 mg kg^−1^, NS-398 10 mg kg^−1^. Starting on day 7, subcutaneous tumour size was determined by measuring the longest and shortest diameters of the tumour at 2–3 days intervals. Tumour volume (mm^3^) was calculated by a standard formula: volume=(the shortest diameter)^2^ × (the longest diameter) × 0.5. Tumour weight was also measured on day 18 after tumours were removed from the killed mice. Tumour tissue was frozen in liquid nitrogen and stored at −70°C.

For the liver metastasis tumour model, 2 × 10^4^ cells ml^−1^ MC-26 cells were injected into the subsplenic capsule of 6–10-week-old BALB/*c* mice under intraperitoneal anaesthesia using pentobarbital (65 mg kg^−1^ body weight) on day 0. After full recovery from surgery, mice were randomly divided into three groups (*n*=12 per group): vehicle (DMSO), NS-398 10 mg kg^−1^, and NS-398 100 mg kg^−1^. Mice were administered with NS-398 once a day by gavage, starting on day 1. Six mice in each group were killed on day 10 and another six mice on day 14. The incident rates of liver metastases were recorded.

### Western blot hybridisation

Mouse cyclin D1 and COX-2 monoclonal antibodies were purchased from BD transduction laboratories (Lexington, KY, USA). To extract the protein, MC 26 cells were harvested and lysed in RIPA buffer (PBS, 1% NP-40, 0.5% sodium deoxycholate, 0.1% SDS, 100 ng ml^−1^ PMSF, 66 ng ml^−1^ aprotinin). To extract protein from tissues, 0.1 g tumour tissue was put in 2.0 ml of cold protein extract buffer (RIPA buffer) and homogenised for 1 min with Polytron-Aggregate (Kinmatica, Luzern, Switzerland). After the removal of cellular debris by centrifugation, total protein extracted from cells or tissues was determined by BCA protein assay (Pierce chemical, Rockford, IL, USA). Protein was mixed with gel-loading buffer (50 mM Tris pH 6.8, 2% SDS, 10% glycerol, 2% 2-mercaptoethanol, 0.1% bromphenol blue) and heated for 10 min at 100°C. Samples containing 5–20 *μ*g protein were loaded onto 10–12% SDS–PAGE gel, and then electrophoretically transferred to polyvinylidene difluoride membrane in a transfer buffer (25 mM Tris, 190 mM glycine, 20% methanol). The blots were blocked with 7% dry milk for 1 h at room temperature and incubated with the first antibody overnight. The blots were then washed three times for 15 min each in Tris-buffered saline containing 0.05% tween-20. The blots were further incubated with the anti-mouse IgG antibody (Sigma Chemical Co., St Louis, MO, USA) for 1 h at room temperature. After washing three times, blots were incubated with luminous ECL reagent (Pierce chemical, Rockford, IL, USA) for 10–120 s and exposed to Kodak X-ray film. Protein bands were identified by protein size and positive control provided by BD transduction laboratories (Lexington, KY, USA). The amount of protein expression was quantified by densitometry.

### Immunohistochemistry

Proliferating cell nuclear antigen (PCNA) monocolonal antibodies were purchased from BD transduction laboratories. Paraffin-embedded specimens were deparaffinised and incubated with PCNA antibody for 2 h at 37°C. The specimens were then incubated with secondary antibody, anti-mouse IgG, for 1 h at 37°C, and stained by the avidin–biotin peroxide complex (ABC) method using an ABC staining system (Santa Cruz Biotech, Santa Cruz, CA, USA). They were visualised by 3,3′-diaminobenzidine (DAB) and counterstained with haematoxylin. To confirm the specificity of the mouse PCNA antibody, human tonsil specimens was used as a positive control. The PCNA index was evaluated by counting the number of PCNA-positive-staining cells per 500 tumour cells: PCNA index=(numbers of PCNA-positive-staining cells/500 cells counted) × 100%.

### Statistical analysis

One-way ANOVA was performed for comparing the tumour volume and weight, PCNA index and densitometric values of Western blot bands among different animal groups, followed by Tukey's procedure for pairwise comparison. Incident rates of liver metastatsis were analysed using the Fisher's exact test. Statistical significance was assigned if *P*<0.05.

## RESULTS

### COX-2 expression and prostaglandin levels following treatment with NS-398

**Figure 1:**
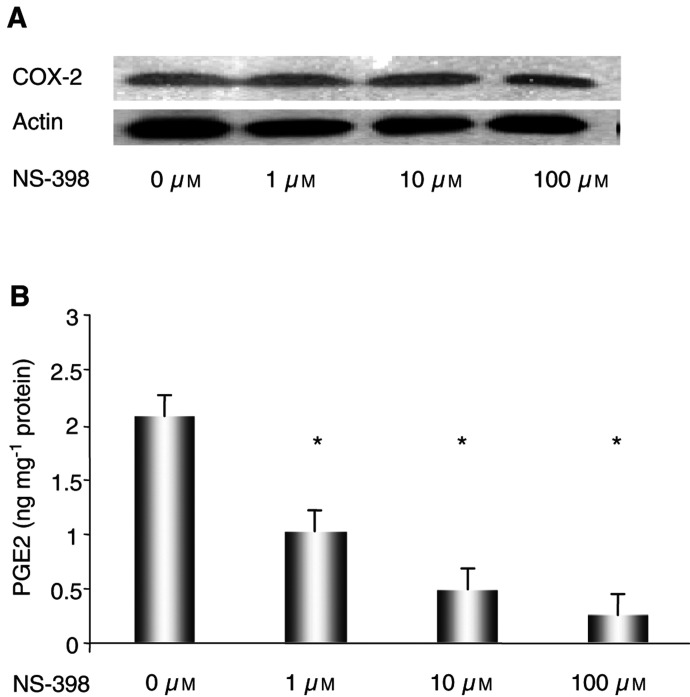
(**A**) Western blot of COX-2 protein and (**B**) quantification of its product, PGE_2_. MC-26 cells were maintained in DMEM media supplemented with 10% foetal calf serum. Cells (100 000 ml^−1^) were seeded for 24 h, followed by the addition of different concentrations of NS-398. Total protein was extracted and Western blot hybridisation performed for COX-2 expression. PGE_2_ was measured using ELISA, as described in Materials and methods section. Measurements were made in triplicate in three separate experiments, and data are depicted as the mean±standard error (s.e.). ^*^P<0.01, compared to control (vehicle).

Although NS-398 has been shown to decrease the activity of COX-2 in some CRC cell lines, no significant changes in COX-2 protein expression were detected in mouse colorectal adenocarcinoma cell line MC-26 after treatment with NS-398 (1, 10 and 100 *μ*M) for 24 h (Figure 1A

). In contrast, concentration-dependent decreases in PGE_2_, a major product of the arachidonic acid pathway, were demonstrated after 24 h of incubation with NS-398. Compared with vehicle incubation (2.08±0.35 ng mg^−1^ total protein), PGE_2_ content in cells treated with 1, 10 and 100 *μ*M NS-398 were diminished by approximately 50% (1.00±0.20 ng mg^−1^), 80% (0.48±0.03 ng mg^−1^) and 90% (0.25±0.07 ng mg^−1^), respectively (Figure 1B).

### Cell proliferation and tumour growth *in vivo*

**Figure 2:**
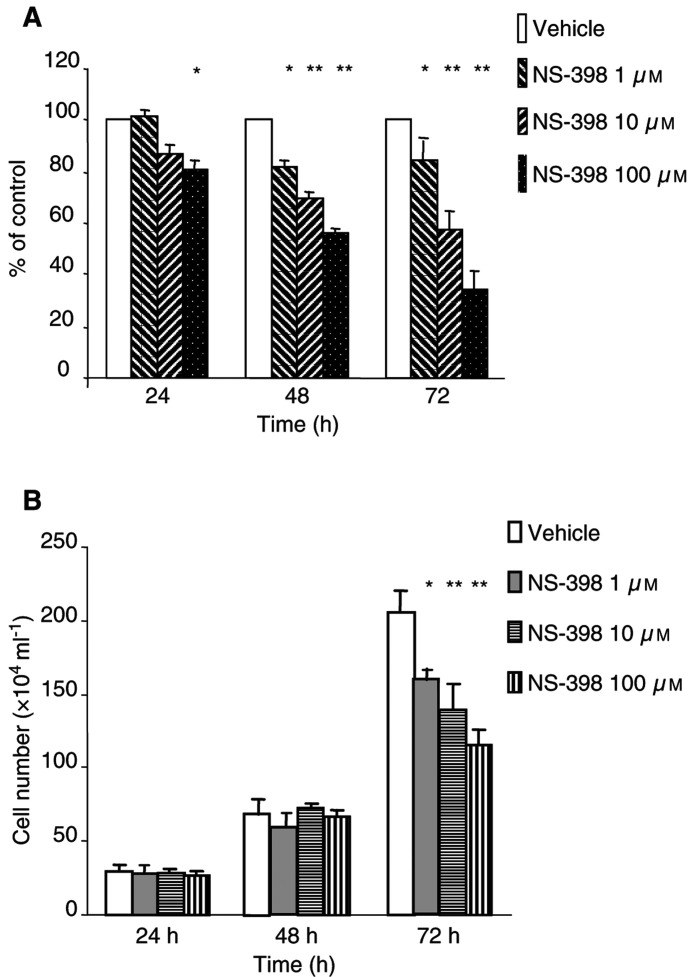
Effect of NS-398 on cellular proliferation. (**A**) DNA synthesis was measured by [^3^H]-thymidine incorporation into cellular DNA. [^3^H]-thymidine 1 *μ*Ci ml^−1^ was added and allowed to label for 6 h at 37°C. Cell lysates were analysed in a liquid scintillation counter. Data are expressed as the percentage of control (vehicle)±s.e. (**B**) Cells (10^4^ ml^−1^) were seeded onto six-well plates for 24 h, followed by the addition of different concentrations of NS-398. Cells were harvested and then cell numbers were manually counted under the microscope. ^*^P<0.05, ^**^P<0.01, compared to control (vehicle).

**Figure 3:**
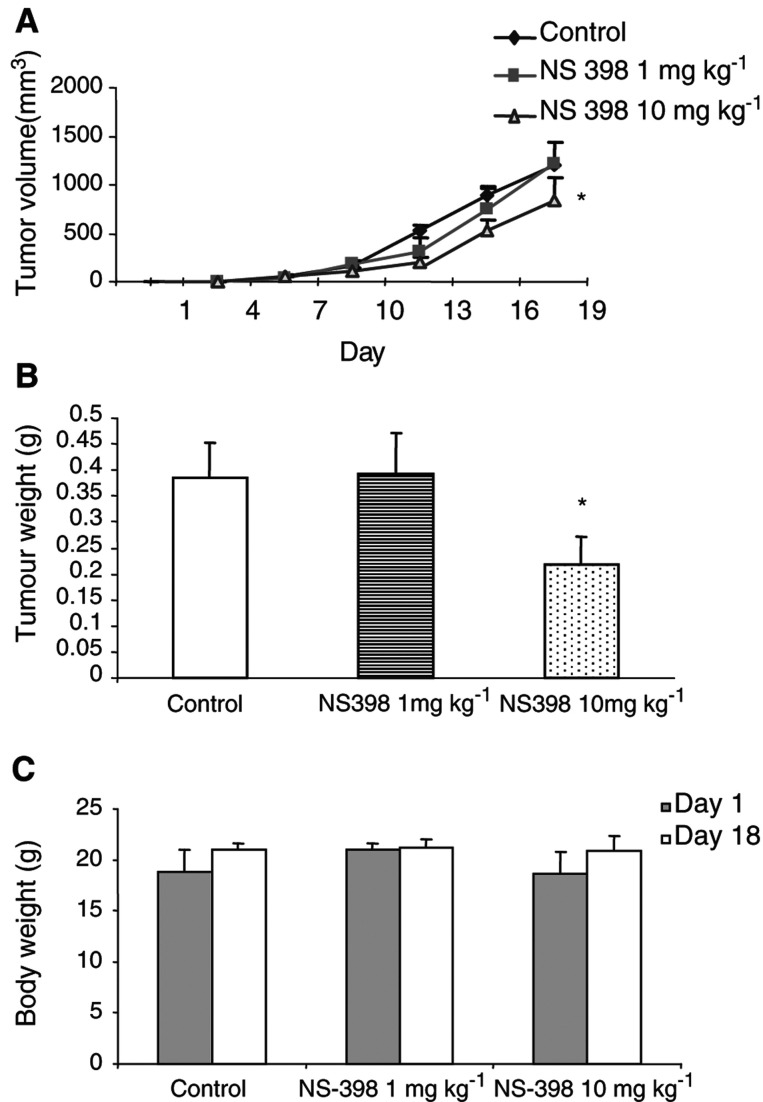
(**A**) Tumour growth (volume) *in vivo* over time and (**B**) tumour weight and (**C**) body weight after excision on day 18. Tumour volume (mm^3^) was determined by measuring the longest and shortest diameter of the tumour, and was calculated by a standard formula: volume=(the shortest diameter)^2^ × (the longest diameter) × 0.5. Tumour weight was measured on day 18, after excision from the killed mice. Data are expressed as the mean±s.e. ^*^P<0.05, compared to control (vehicle).

The addition of NS-398 to MC-26 cells led to a concentration-dependent decrease in [^3^H]-thymidine incorporation. NS-398 1, 10 and 100 *μ*M decreased cell proliferation to 84.3, 57.2 and 34.6% at 72 h, respectively, compared to cells incubated in the absence of NS-398 (Figure 2A

). These results had been confirmed by cell number counting, showing that significant cell number reduction occurred at 72 h after NS-398 treatment (Figure 2B). Figures 3A and B

depict the effects of NS-398 on subcutaneous tumour volume and tumour weight at the end of the study (day 18). Although low-dose (1 mg kg^−1^) NS-398 did not affect subcutaneous tumour growth compared with control animals (tumour volume: 1208.2±223.6 *vs* 1220.2±224.0 mm^3^; tumour weight: 0.392±0.08 *vs* 0.384±0.07 g, *P*>0.05), high-dose (10 mg kg^−1^) NS-398 significantly decreased subcutaneous tumour growth (tumour volume: 843.3±226.8 mm^3^; tumour weight: 0.218±0.05 g, both *P*<0.05). As shown in Figure 3C, no significant changes in body weight were detected among these groups.

**Figure 4:**
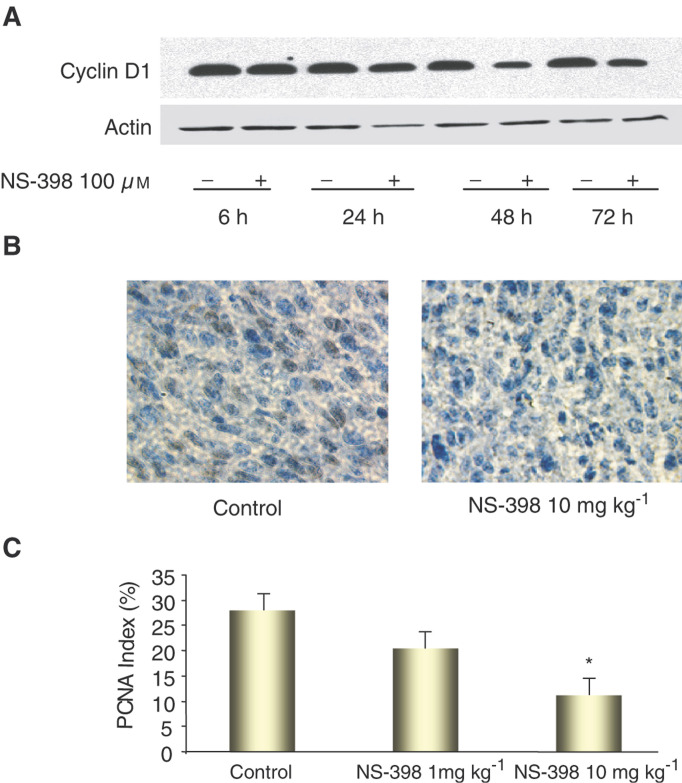
(**A**) Effect of NS-398 on cyclin D1 protein expression in cells over time, (**B**) representative photomicrograph of PCNA staining of tumour tissue, and (**C**) PCNA index. Cyclin D1 protein was detected by Western blot hybridisation, and PCNA was determined by immunohistochemistry. PCNA index was calculated by counting the number of PCNA-positive-staining cells per 500 tumour cells: PCNA index=(numbers of PCNA-positive cells/500 cells counted) × 100. ^*^P<0.05, compared to control.

To further assess the inhibitory effects of NS-398 on cell proliferation and tumour growth, we measured cyclin D_1_ and PCNA levels using Western blot hybridisation and immunohistochemistry. Cyclin D_1_ is one of the key proteins involved in cell cycle regulation in normal cells (Hunter and Pines, 1994; Sherr, 1996). In the G_1_ (resting) phase of the cell cycle, cyclin D_1_ along with its cyclin-dependent kinase (CDK) partner, is responsible for transition to the S (DNA synthesis) phase. Overexpression of cyclin D_1_ releases a cell from its normal control and causes transformation to a malignant phenotype (Motokura and Arnold, 1993; Bartkova *et al*, 1994; Arber *et al*, 1996; Sutter *et al*, 1997). PCNA functions as an auxiliary protein to DNA polymerase-*γ* and as a co-factor in DNA synthesis. The determination of PCNA represents one of the most reliable methods for evaluating proliferation in cells and tissues (Prosperi, 1997). In the present study, consistent with the cell proliferation and tumour growth, cyclin D_1_ levels (normalised to actin protein) were decreased after MC-26 cells were incubated in the presence of NS-398 (100 *μ*M) for 48 and 72 h (Figure 4A

). The PCNA index was also significantly decreased in mice treated with NS-398 10 mg kg^−1^, compared with mice administered vehicle only (27.0±3.2 *vs* 10.8±2.6%, *P*<0.01) (Figure 4B, C).

### Effects of NS-398 on cell invasion potential and liver metastasis

**Figure 5:**
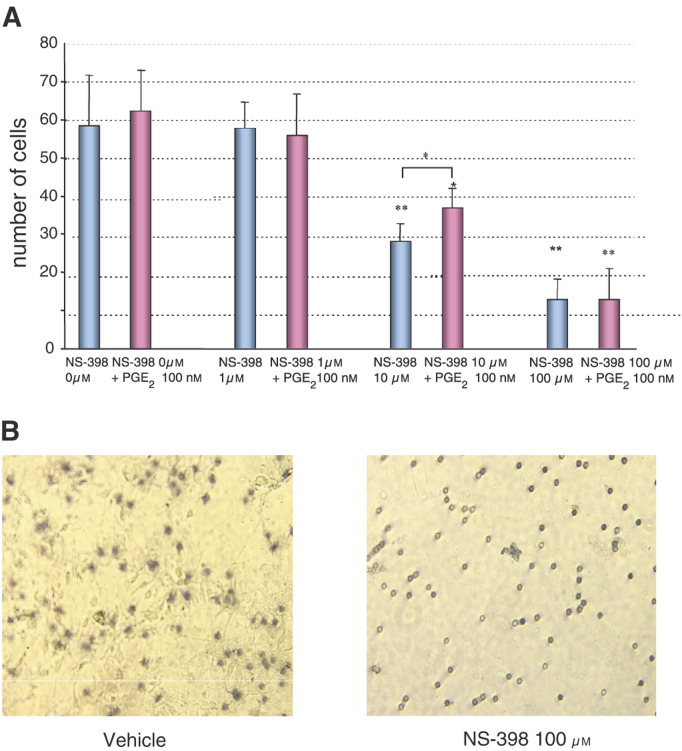
Effect of NS-398 on cell invasion/migration *in vitro*. Cell invasion/migration assay was performed using the Matrigel invasive chamber system, as described in Materials and methods section. (**A**) Number of cells on the undersurface of filters after 24 h in the presence of NS-398 alone, or in combination with PGE2 100 nM. (**B**) Representative photomicrograph of cells on the undersurface of filters after 24 h in the presence of NS-398 100 *μ*M or vehicle. ^*^P<0.05, ^**^P< 0.01, compared to NS-398 0 *μ*M.

To quantify cell invasion, we employed a Matrigel invasion chamber assay. Matrigel is a solubilised basement membrane preparation containing extracellular components such as laminin, collagen type IV, heparin sulphate proteoglycan, and growth factors such as TGF and basic FGF. The layer of Matrigel Matrix simulates a reconstituted basement membrane *in vitro*. To perform these experiments, an invasion assay was established, that included upper and lower chambers separated by a filter, onto which the cells are trapped and can be counted. The bottom of the upper chamber was coated with Matrigel. Highly invasive cells are able to degrade the extracellular matrix components and migrate through the layer of Matrigel to the other side of filter by activating various proteinases. The lower chamber was filled with serum-free conditioned 3T3 fibroblast medium, to act as a chemoattractant. Cells pretreated with NS-398 or vehicle were loaded into the upper chamber, and, following a 24-h incubation period, the number of cells attached to the filters below the Matrigel layer was counted. As shown in Figure 5

, 1–100 *μ*M NS-398 inhibited cell invasion/migration through the Matrigel extracellular matrix components at 24 h in a concentration-dependent reduction, with maximum inhibition of 78% of control (13.0±5.4 *vs* 58.5±13.1 cells per microscopic field under control conditions, *P*<0.01). Since cell number did not significantly decrease at 24 h after NS-398 treatment (Figure 5), the inhibitory effect of NS-398 at 24 h may occur as a result of reduced cell-invasive capacity rather than a reduction in cell number. The addition of exogenous PGE_2_ partially attenuated the inhibition of cell invasion produced by 10 *μ*M NS-398, but not in cells treated with 100 *μ*M NS-398.

**Figure 6:**
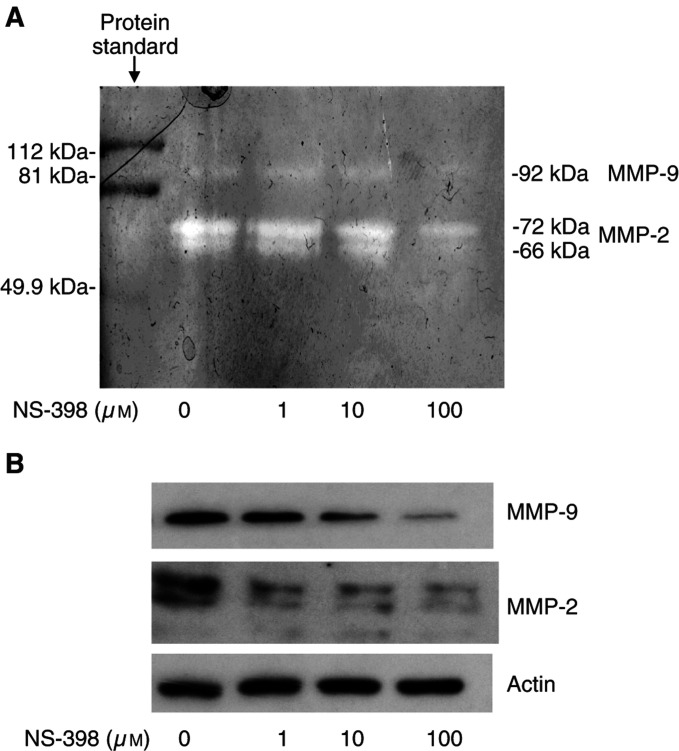
(**A**) Activity of MMP-2 and -9 in NS-398-treated cells using gelatin zymography and (**B**) protein expression of MMP-2 and -9 using Western blot hybridisation, as described in Materials and methods section. MC-26 cells were cultured without and with increasing concentrations of NS-398 for 24 h.

To further explore the molecular mechanisms by which NS-398 decreased the invasive capacity of MC-26 cells, we measured MMP concentrations. Metalloproteinases consist of a family of nine or more highly homologous Zn^2+^ peptidases that collectively cleave the constituents of extracellular matrix and facilitate tumour cell invasion and migration. Metalloproteinases play an important role in tumour invasion and metastasis (Curran and Murray, 2000; Nelson *et al*, 2000). Within the MMP family, MMP-2 and -9, also called gelatinases or type-IV collagenases, have been implicated to play a significant proteolytic role in CRC invasion and metastasis. As seen in Figure 6

, after the incubation of MC-26 cells with 100 *μ*M NS-398 for 24 h, both protein expression (Western blot) and enzyme activity (zymography) of MMP-2 and -9 enzymes were decreased by approximately 25–30% (*P*<0.05).

**Figure 7:**
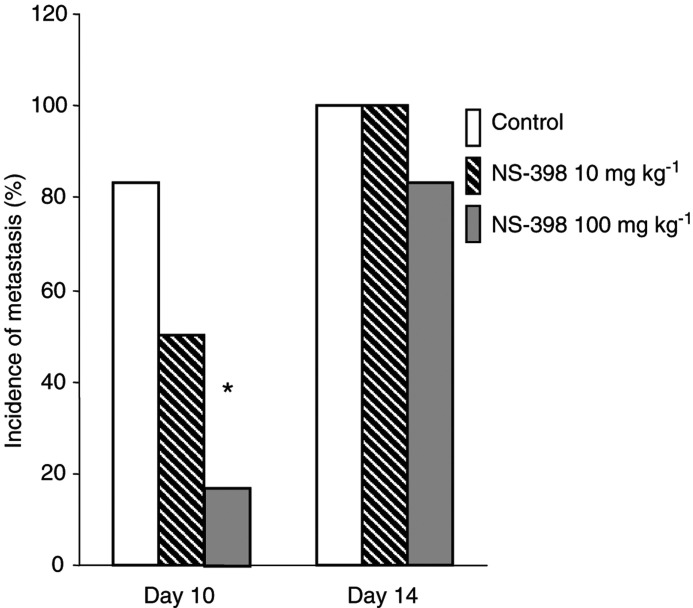
Effect of NS-398 on liver metastasis. A mouse CRC liver metastasis model was established by splenic subcapsule tumour cell injection. Mice were administered vehicle or NS-398 (10 or 100 mg kg^−1^) once daily by gavage. On days 10 and 14, the incidence of liver metastasis was recorded. ^*^P<0.05, compared to control.

The liver is the major target organ for CRC metastasis. We established a CRC liver metastasis model by injecting tumour cells into the splenic subcapsule. Visible metastatic lesions in the liver were detected by day 7 after cell inoculation. On day 10, the incidence of liver metastasis in the NS-398-treated group (100 mg kg^−1^ day^−1^) was significantly lesser than that in control group (one out of six *vs* five out of six, *P*<0.05). However, on day 14, liver metastasis occurred in 100% (six out of six) in control and 83.33% (five out of six) in the NS-398-treated group (NS-398 100 mg kg^−1^ day^−1^, *P*>0.05). These results indicate that NS-398 delayed, but did not prevent, liver metastasis (Figure 7

).

## DISCUSSION

COX-2 overexpression has been demonstrated in ∼85% of cases of primary CRC. Overexpression of COX-2 appears to alter the phenotype of intestinal epithelial cells and increases their carcinogenic potential. A number of epidemiological studies have shown that the prolonged use of aspirin and other nonsteroidal anti-inflammatory drugs (NSAIDs) can reduce the relative risk of CRC (Giovannucci *et al*, 1994; Smalley *et al*, 1999). For example, a recent population-based survey in >100 000 individuals 65 years of age and older found that the long-term use of NSAIDs diminished the risk of CRC by ∼50% (Giovannucci *et al*, 1994; Smalley *et al*, 1999). Another large population-based survey reported that the use of NSAIDs significantly reduced the risk of both gastric and oesophageal carcinoma (Farrow *et al*, 1998). Although the precise mechanisms by which NSAIDs, and COX-2 selective inhibitors in particular, modulate tumour growth have not been elucidated, it appears that cell cycle arrest and apoptosis may play a critical role (Chang and Weng, 2001; Grosch *et al*, 2001; Cheng *et al*, 2002; Joe *et al*, 2002; Kundu *et al*, 2002; Toyoshima *et al*, 2002). Alternatively, Sawaoka *et al* (1998) and Tsujii *et al* (Tsujii and DuBois, 1995; Sawaoka *et al*, 1998; Tsujii *et al*, 1998) have suggested that a decrease in angiogenesis may account for the inhibitory properties of COX-2 selective NSAIDs on tumour growth. Early studies demonstrated that sulindac was effective in reducing the number of colonic polyps in individuals with familial adenomatous polyposis (FAP), presumably by decreasing the activity of COX (Waddell and Loughry, 1983). Selective COX-2 inhibitors share the antitumour properties of nonselective NSAIDs like sulindac, but are associated with far fewer adverse GI events (Wolfe, 1998; Wolfe *et al*, 1999). Cyclooxygenase-2 selective inhibitors have been shown to inhibit the growth of tumour cells *in vitro* (Sheng *et al*, 1997). They also reduce the development of precancerous adenomatous polyps in the *Apc*^Δ716^ knockout mouse, a model of human hereditary familial adenomatous polyposis, and in the azoxymethane rat model, which produces benign and malignant colorectal adenomatous polyps analogous to nonhereditary spontaneous development of colorectal adenocarcinoma (Williamson *et al*, 1978; Oshima *et al*, 1996; Singh *et al*, 2000).

In December 1999, the US Food and Drug Administration approved the use of the COX-2 selective NSAID celecoxib in individuals with FAP, both for the regression and reduction of polyps. While FAP serves as an excellent model for CRC, it is a rare condition, accounting for <1% of all cases. The present therapy for the treatment of advanced CRC is not only quite toxic, but also largely ineffective. A previous *in vitro* study using cell lines of CRC origin by Tsujii *et al* (1997) suggested that COX-2 inhibition may be beneficial in preventing the expression of genes associated with metastatic progression. Furthermore, a recent study by Tomozawa *et al* (1999) demonstrated that the COX-2 selective inhibitor JTE-522 might decrease the rate of haematogenous metastasis of CRC.

Many NSAIDs have been found to inhibit cell division and alter cell cycle distribution in culture colon cancer cells (Xu *et al*, 1999; Grosch *et al*, 2001; Kundu *et al*, 2002). In the present study, cell proliferation was inhibited by NS-398, a COX-2 selective inhibitor, in a concentration-dependent manner, with a maximum inhibition of 34.6% of control values. In the subcutaneous colon tumour models, mice treated with 10 mg kg^−1^ NS-398 displayed approximately 50–70% of the tumour burden compared to control animals. To confirm the antiproliferative effect of NS-398, we examined two proliferation-related molecules: cyclin D1 and PCNA. Previous studies have demonstrated that cyclin D1 is increased in adenomatous polyps and in both sporadic and familial forms of CRC (Motokura and Arnold, 1993; Bartkova *et al*, 1994; Arber *et al*, 1996; Sutter *et al*, 1997). The synthesis and expression of PCNA are enhanced in proliferating cells, including those that are tumour-derived. Consistent with the cell proliferation and animal tumour growth data observed, cyclin D1 and PCNA index were significantly decreased by NS-398 both *in vitro* and *in vivo*.

Tumour metastasis is a complex multifactorial and multistep biological process. Tumour cell invasive potential, host microenviroment and interactions between host and tumour cells are among the factors influencing the establishment and development of metastatic lesions (Woodhouse *et al*, 1997). Cyclooxygenase-2 overexpression has been associated with increased adhesion to the extracellular matrix and the inhibition of apoptosis (Tsujii and DuBois, 1995). Cyclooxygenase-2 also leads to alterations in the invasive potential of CRC cells (Tsujii *et al*, 1997). Metastatic CRC cells from liver and primary CRC tissue exhibit much higher levels of COX-2 than the corresponding adjacent normal mucosa from the same patient. Among patients with relatively high COX-2 expression in the primary tumour, almost all were found to exhibit even higher levels of COX-2 in their hepatic metastases (Chen *et al*, 2001). The precise mechanism underlining the roles of COX-2 in the metastasis process is unknown. It has been reported that proteolysis of the extracellular matrix and angiogenesis are involved in the process (Tsujii and DuBois, 1995; Tsujii *et al*, 1997, 1998). One of the major steps in the process of cancer cell invasion is proteolysis of the extracellular matrix, especially the basement membrane, including fibronectin, laminin, type IV collagen and proteoglycans (Coussens *et al*, 2002; Vihinen and Kahari, 2002). Using reconstituted basement membrane Matrigel *in vitro*, we found that NS-398 significantly inhibited the capacity of cell invasion and migration through the extracellular matrix by inactivating the activities of MMP-2 and -9. Metalloproteinase inhibition could thus account for one of the molecular mechanisms involved in mediating the anti-invasive properties of NS-398. We also found that the addition of exogenous PGE_2_ partially attenuated the inhibition effect of NS-398 at 10 *μ*M, but not with 100 *μ*M, indicating that the invasion/migration-inhibitory effects of NS-398 might also be modulated through COX-independent pathways.

To determine whether NS-398 possessed any inhibitory effects on the development of liver metastasis, we utilised an animal model that generates metastasis by the injection of MC-26 cells into the splenic subcapsule. Although we did observe a decrease in subcutaneous tumour growth in response to the administration of 10 mg kg^−1^ NS-398 (Figure 3), this same dose had no significant effect on liver metastasis. When administered by gavage at a daily dose of 100 mg kg^−1^, however, the rate of metastasis was significantly diminished (Figure 7). The mechanisms accounting for the higher dose requirements are not known, but may be due, at least in part, to the more abundant blood supply in the spleen compared to the subcutaneous space or alternatively to differences in the cellular and molecular mechanisms responsible for mediating the effects of COX-2 inhibition on tumour growth and metastasis. NS-398 at the 100 mg kg^−1^ dose decreased the development of liver metastasis on day 10, but not on day 14, indicating that NS-398 appears to retard, but not prevent, the early formation of liver metastasis using this model.

In conclusion, the expression and activity of COX-2 appears to be associated with the proliferative and invasive properties of CRC. Cyclooxygenase inhibition suppresses tumour cell growth and invasion, and retards the formation of liver metastasis in a mouse CRC model by multiple cellular and molecular mechanisms. Further studies are warranted to evaluate the possibility that COX-2 inhibition be considered as part of the treatment regimen for advanced CRC.
